# Ketogenic diet ameliorates lipid dysregulation in type 2 diabetic mice by downregulating hepatic pescadillo 1

**DOI:** 10.1186/s10020-021-00429-6

**Published:** 2022-01-03

**Authors:** Jielin Zhou, Yao Lu, Yajing Jia, Jing Lu, Zhengxuan Jiang, Keyang Chen

**Affiliations:** 1 Department of Nutrition and Food Hygiene, School of Public Health, Anhui Medical University, Hefei, 230032 Anhui China; 2 Department of Anesthesiology, The First Affiliated Hospital, Anhui Medical University, Hefei, 230032 Anhui China; 3 Department of Health Inspection and Quarantine, School of Public Health, Anhui Medical University, Hefei, 230032 Anhui China; 4 Department of Ophthalmology, The Second Affiliated Hospital, Anhui Medical University, Hefei, 230021 Anhui China

**Keywords:** Ketogenic diet (KD), β-Hydroxybutyrate (β-HB), PES1, Lipid metabolism, Inflammation

## Abstract

**Background:**

Previous reports implied a possible link between PES1 and lipid metabolism. However, the role of PES1 in regulating T2DM related lipid metabolism and the effect of ketogenic diet (KD) on PES1 have not been reported. The aim of present study is to explore the role of PES1 in effects of KD on diabetic mice and its mediated mechanism.

**Methods:**

Male C57BL/6J and KK*A*^y^ mice were fed with standard diet (SD) and KD, respectively. Simultaneously, McArdle 7777 cells were treated by β-hydroxybutyric acid (β-HB), *Pes1* siRNA or *Pes1* overexpression plasmid, respectively. Additionally, liver-conditional knockout (CKO) of *Pes1 *in vivo was applied.

**Results:**

Hepatic PES1 expression in diabetic mice was markedly increased, which was suppressed by KD feeding with an accompanying reduction of hepatic and plasma triglycerides (TG). In mice with CKO of *Pes1*, the protein levels of p300, SREBP1c, FASN, SCD1, Caspase1, NLRP3 and GSDMD were dramatically downregulated in livers, and the plasma and hepatic TG, IL-1β and IL-18 were decreased as well. The similar outcomes were also observed in β-HB and *Pes1* knockdown treated hepatocytes. By contrast, *Pes1* overexpression in cultured hepatocytes showed that these levels were significantly enhanced, which were, however reduced under β-HB treatment. Mechanistically, we discovered that β-HB decreased CHOP binding to the *Pes1* promoters, resulting in the downregulation of PES1, thereby reducing PES1 binding to *p300* and *Caspase1* promoters. The inhibition of p300 and Caspase1 expression elicited the dramatic suppression of acetylation of SREBP1c via its interaction with p300, and the decreased GSDMD levels. Besides, knockdown of *Caspase1* also alleviated the TG levels in cultured hepatocytes.

**Conclusion:**

KD may improve lipid dysregulation in type 2 diabetic mice by downregulating hepatic PES1 expression.

**Supplementary Information:**

The online version contains supplementary material available at 10.1186/s10020-021-00429-6.

## Introduction

Type 2 diabetes mellitus (T2DM), characterized by insulin resistance (IR) and pancreatic β-cell dysfunction, is a heterogeneous metabolic disorder (Magliano et al. 2020). Compelling evidence showed that T2DM was accompanied by hypertriglyceridemia-based lipid dysregulation (Zheng et al. 2018; Xu et al. 2018), one of the main factors leading to atherosclerosis and even death (McGuire et al. 2016).

Recently, an interesting dietary pattern, namely ketogenic diet (KD), has been proposed to attain a remarkable decline of hyperglycemia in T2DM (Abbasi 2018). KD is typically characterized by low carbohydrates (5 to 10% of total caloric intake) and high fat consumption (more than 70% of total caloric intake) (Castellana et al. 2020). Additionally, it was reportedly associated with the remission of numerous adverse health outcomes such as obesity, inflammation, cardiovascular disease and cancer (Gentile and Weir 2018; Allen et al. 2014; Yancy et al. 2019; Augustin et al. 2018; Watanabe et al. 2020). One study has documented that the KD could alleviate the hyperglycemia in T2DM through producing ketone bodies that approximately consist of 78% β-hydroxybutyric acid (β-HB), 20% acetoacetic acid and 2% acetone (Abbasi 2018; Newman and Verdin 2017). Due to the high fat content in KD, the bad outcomes by the long-term consumption of KD, including no significant weight loss, lipid accumulation, and liver fibrosis, were also reported in some studies (Ellenbroek et al. 2014; Zhang et al. 2016). Nevertheless, the molecular mechanisms underlying the impact of KD have not been satisfactorily explored.

Currently, we unexpectedly observed that hepatic pescadillo 1 (PES1) expression in T2DM mice were markedly elevated. PES1, also known as pescadillo ribosomal biogenesis factor 1, or NOP7, or YPH1, was originally found in zebrafish embryos (Allende et al. 1996). It is evolutionarily highly conserved and forms a complex with BOP1 and WDR12 (PeBoW complex), which is essential for the assembly of 60S ribosomal subunits, DNA replication, cell cycle progression, etc. (Du and Stillman 2002; Cheng et al. 2019). PES1 is aberrantly upregulated in various malignant tumors such as breast, ovarian and colon cancers, as well as hepatocellular carcinoma (Cheng et al. 2012; Fan et al. 2018; Li et al. 2013). A considerable number of publications have reported that T2DM with lipid dysregulation is associated with increased incidence and mortality from many cancers (Shlomai et al. 2016; Klil-Drori et al. 2017). And one published study discovered that circular antisense non-coding RNA in the INK4 locus (circANRIL), a prototype of circRNA regulating ribosome biogenesis and conferring atheroprotection, acts as a molecular inhibitor of PES1 by binding at the C-terminal domain of PES1 (Holdt et al. 2016). Collectively, these reports suggested the possible link of PES1 to lipid metabolism under T2DM condition.

In this study, we aimed to evaluate the effects of KD on diabetic mice or of β-HB on liver cells via downregulating PES1 expression, which may be associated with the amelioration of lipid metabolism in vivo and in vitro. Thus, a novel mechanism of PES1 mediated lipid metabolism under T2DM condition affected by KD intervention would be probed. Our current study may provide a novel therapeutic target for T2DM management and treatment by KD or PES1 inhibitor.

## Materials and methods

### Reagents

β-HB (166898) was bought from Sigma Chemical Co (St. Louis, MO). Triglycerides (TG), total cholesterol (TC), alanine aminotransferase (ALT), aspartate aminotransferase (AST) and β-HB assay kits were from Nanjing Jiancheng Bioengineering Institute (Jiangsu, China). The ELISA kit for insulin was obtained from Jianglai Corp (Shanghai, China). Flag-tag (20543-1-AP), β-actin (66009-1-Ig) and Caspase1 (22915-1-AP) antibodies for western bolting, and PES1 (13553-1-AP) antibody for immunofluorescence were obtained from Proteintech, Chicago, USA. PES-1 (NBP2-55211) antibody for western bolting and SREBP1c antibody (NB600-582) for immunofluorescence were bought from Novus Biologicals. SREBP1c (191857), SCD1 (ab19862) and SREBP2 (ab155017) antibodies for western bolting were purchased from Abcam. FASN (3180S) antibody for western bolting was from Cell Signaling Technology (Beverley, MA). NLRP3 (DF7438) and GSDMD (AF4012) antibodies for western bolting were from Affinity. CHOP (sc-7351), p300 (sc-48343) and Ac-SREBP1c (Acetylated-SREBP1c, sc-13551 AC) antibodies were bought from Santa Cruz Biotechnology. RNAex Pro Reagent (AG21102), reverse transcription kits (AG11707), SYBR Green qPCR SuperMix (AG11718) were bought from Accurate Biology. Cell counting kit-8 (CCK-8) (C0005) was from Target Mol (Shanghai, China). Chromatin immunoprecipitation (ChIP) assay kit (P2078) was purchased from Beyotime. Rat *Pes1* and *Caspase1* short interfering RNA (siRNA) and *Pes1* overexpression plasmid were purchased from GENERAL BIOL (Anhui, China).

### Animal grouping

Five-week-old male C57BL/6J and KK*A*^y^ (KK.Cg-Ay/J) mice were from Beijing Vital River. The housing unit was maintained at constant temperature 22–25 °C and 50–60% relative humidity with a 12/12-h light/dark cycle and free access to tap water. KK*A*^y^ mice were fed with commercial high-fat diet (BEIJING HFK Bio-Technology Co., Ltd., Beijing, China) ad libitum for 4 weeks to increase their plasma glucose levels. After 4 weeks, all mice were divided into four groups (10–12 mice per group), including C57BL/6J fed with standard diet (SD, LAD3001G, Trophic Animal Feed High-Tech Co., Ltd, China) (C57BL/6J-SD) and ketogenic diet (KD, TP 201455, Trophic Animal Feed High-Tech Co., Ltd, China) (C57BL/6J-KD), and KK*A*^y^ fed with SD (KK*A*^y^-SD) and KD (KK*A*^y^-KD). The composition of the diet was listed in Additional file 1: Table S1. All mice were fed for 16 weeks. The food and water intakes were measured three times a week and taken by the average. Body weight and fasting plasma glucose (fasted for 6–8 h) were recorded once a week. Feeding efficiency was calculated as body weight gain (mg) per kcal food consumed. Our procedures on mice obeyed the guidelines for humane treatment set by the Association of Laboratory Animal Sciences at Anhui Medical University.

### GTT and ITT tests

Fasting glucose was measured weekly by Roche blood glucose meter. Glucose tolerance test (GTT) and insulin tolerance test (ITT) were performed before the mice were sacrificed, the concentrations of glucose and insulin for intraperitoneal injection were 2 mg/g body weight and 0.7 mU/g body weight, respectively. Blood glucose samples were tested at 0, 30, 60, 90, 120, 150 min and 0, 20, 40, 60, 80, 100, 120 min, respectively. Area-under-curve (AUC) was calculated by the trapezoid rule.

### Murine tissue and blood sample collection

By the end of experiment, all fasted mice were euthanized with 10% chloral hydrate and then killed by cervical dislocation to obtain tissues and blood samples. Blood was immediately centrifuged at 3000 rpm for 10 min and the levels of insulin in plasma were detected by enzyme linked immunosorbent assay (ELISA) kits. The homeostatic model assessment of insulin resistance (HOMA-IR) index was calculated using the following formula: fasting insulin levels (mIU/L) × fasting glucose levels (mmol/L)/22.5. The remaining plasma was stored at − 80 °C for later analysis. The liver tissues were rinsed with cold phosphate-buffered saline (PBS). Small portions of liver tissues were fixed in 4% paraformaldehyde solution for oil red O and hematoxylin–eosin (H&E) staining respectively. Then two-third of liver per mice was frozen in liquid nitrogen immediately and kept at − 80 °C for immunoblotting and biochemical analysis. The remaining livers were preserved in RNAlater for qRT-PCR test. TC, TG, ALT and AST were determined using enzymatic kits in accordance with the manufacturer's instruction.

### Cell culture

McArdle 7777 rat hepatoma cells were obtained from the American Type Culture Collection (Manassas, VA, USA, cat# CRL-1601). McArdle 7777 cells were cultured in DMEM with high glucose concentration (25 mmol/L), supplemented with 10% FBS and 1% penicillin/streptomycin in an incubation chamber with 5% CO_2_ at 37 ℃. The concentration and time of β-hydroxybutyric acid (β-HB) treatment were selected depending on cell counting kit-8 (CCK-8) test. Specifically, McArdle liver cells were seeded in the 96-well plates for 24 h. Then by the β-HB treatment with a series of concentrations (0, 0.25, 1, 2, 4 mM) and times (0, 12, 24, 48 h), CCK-8 reagents (10 µL) were added into the treated cells. Next, the cells were incubated for 1–4 h at 37 °C. Simultaneously, the medium was treated in the same way. The absorbance at 450 nm was detected by the microplate reader. Cellular viability was calculated by the absorbance values, by which the most suitable treatment concentration and time of β-HB were determined.

### Quantitative real-time PCR

The total RNAs were extracted using RNAex Pro reagent. The genomic DNA was removed using RNase-free DNase. Purified total RNA (1 μg) was reverse-transcribed using reverse transcriptase. qRT-PCR was performed with a Roche Light Cycler 480 System with SYBR Green Super Mix using gene-specific primers (Additional file 1: Table S2). The comparative Ct method was used to determine the amount of target normalized to an endogenous reference (β-Actin) and relative to a calibrator (2^−△△Ct^).

### Immunoblotting

McArdle 7777 cells and liver tissues (30 mg/per sample) were homogenized in lysis buffer, respectively. Total lysates (20 μg per well) were separated electrophoretically by 10% SDS-PAGE and transferred onto PVDF membranes. The membranes were respectively incubated for 24 h with CHOP, PES1, p300, SREBP1c, SREBP2, FASN, SCD1, NLRP3, Caspase 1, GSDMD antibodies at 4 °C. β-Actin was used as a loading control. After being washed 8 min for 4 times with TBST buffer, the membranes were incubated with secondary antibodies for 60 min. The enhanced chemiluminescence reagent was used. The signal was then detected by the digital imaging equipment.

### Immunofluorescence

McArdle 7777 cells were seeded onto 12-well plate containing cell climbing slides for 24 h. After β-HB treatments, cells were washed thrice with PBS and fixed with 4% paraformaldehyde for 20 min. Nonspecific binding sites were blocked with 5% normal bovine serum in PBS. The 12-well plates were incubated with the mixture of primary antibodies including PES1 (1:250) and SREBP1c (1:250) at 4 °C overnight. After PBS washing, the 12-well plates were incubated for 60 min with the Alexa Fluor 488 conjugated secondary antibody (ab150077, Abcam) or Alexa Fluor 568 conjugated secondary antibody (ab175473, Abcam). Cell climbing slides were taken out from the 12-well plate and buckled upside down on the glass slide containing DAPI. All sections were mounted and observed by a confocal microscope (LSM880, Zeiss).

### RNA interference of *Pes1* and *Caspase1*

Rat *Pes1* and *Caspase1* small interfering RNA (siRNA) and Lipofectamine 3000 (L3000008, Invitrogen Life Technologies Crop) were mixed in serum-free medium for 20 min, respectively. Then the mixture was added to the culture medium to transfect McArdle 7777 cells. After 6 h, the cells continue to be cultured in fresh medium for 48 h. Finally, cells were collected for subsequent measuring TG, TC, and running Western Blotting and qRT-PCR. The sequences of *Pes1* siRNA were 5ʹ-UGAAGAAGCGAGAGAAGUATTT-3ʹ (forward) and 5ʹ-UACUUCUCUCGC UUCUUCATT-3ʹ (reverse). The sequences of *Caspase1* siRNA were 5ʹ-AGGAAGA GAUGGAUACAAUTT-3ʹ (forward) and 5ʹ-AUUGUAUCCAUCUCUCUUCCUTT-3ʹ (reverse). The scrambled siRNA control sequences were 5ʹ-UUCUCCGAA CGUGUCACGUTT-3ʹ (forward) and 5ʹ-ACGUGACACGUUCGGAGAATT-3ʹ (reverse).

### Overexpression of *Pes1 *in vitro

The purified plasmid of *Pes1-*flag and Lipofectamine 3000 were mixed in serum-free medium for 20 min. Then the mixture was added into the culture medium to transfect McArdle 7777 cells for 6 h. After being cultured in fresh medium for 24 h, the cells were divided into three groups for different treatments, including negative control (without transfection of *Pes1* plasmid or any reagent), *Pes1-*plasmid and *Pes1-*plasmid plus β-HB. After being cultured for 24 h, cells were collected for subsequent assays of TG, TC, Western Blotting and qRT-PCR.

### Chromatin immunoprecipitation

The binding of CHOP (C/EBP-homologous protein) to the *Pes1* promoter, PES1 binding to the *p300* and *Caspase1* promoters were analyzed by chromatin immunoprecipitation (ChIP). Briefly, cells were crosslinked with 1% formaldehyde for 10 min, neutralized with 125 mM glycine pH 2.5 and washed in PBS. Nuclei were prepared by hypotonic lysis buffer (5 mM Pipes pH 6.8, 85 mM KCl, 0.5% NP40) and centrifugation, and resuspended in SDS lysis buffer, and incubated on ice for 10 min to be fully lysed. Chromatin was sonicated with bioruptor (Diagenode) to be 200–1000 bp average fragment size and cleared by centrifugation. The anti-CHOP, anti-PES1 antibodies or control rabbit IgG (AC005, ABclonal Technology Co., Ltd) was respectively added into an aliquot of 200 μL sonicated lysate, and then 20 μL washed protein A/G-agarose beads (sc-2003, Santa Cruz Biotechnology) was added. The mixture was rotated at 4 °C for 2 h, and then centrifuged at 1000*g* for 1 min at 4 °C to wash the beads. The washed beads were resuspended in TE buffer, vortexed 10 s and boiled for 10 min. The samples and sonicated lysates were treated with 1 μL of 20 mg/mL proteinase K. After being centrifuged at 12,000*g* for 5 min at 4 °C, the digested DNA was used for qRT-PCR assay. Primers for the CHOP binding site in *Pes1* promoter, the PES1 binding site in the rat *p300* and *Caspase1* promoters were as follows: CHOP-*Pes1* binding site (forward) 5ʹ-CTGGTACGTGGGTGCAGTTTGG-3ʹ, CHOP-*Pes1* binding site (reverse) 5ʹ-CACACAGGGATGAACATAAGTGAGAGG-3ʹ; PES1-*p300* binding site (forward) 5′-TCCTCTTGCTGTCTGACTTGTTTGAG-3′, PES1-*p300* binding site (reverse) 5′-AAGATGTTGAGCCTGTTCTCTGAGTTC-3′; PES1-*Caspase*1 binding site (forward) 5′-GGAGCAGGGAAACGATGTATGT GAG-3′, PES1-*Caspase*1 binding site (reverse) 5′- TTGCCCTCAGGATCTTGTCTG TTTAAG-3′.

### Co-immunoprecipitation

Every extracted protein stock solution from cultured cells or liver tissues was divided into two aliquots. The small part was directly used for input assay, and the remaining big portion was pre-cleared with protein A/G-agarose (sc-2003, Santa Cruz Biotechnology) for 2 h. The supernatant after pre-clearance was collected by centrifugation, and further divided into two equal portions. Ac-SREBP1c (acetylated SREBP-1c), p300 and nonimmune IgG (Santa Cruz, sc-2025) antibodies were respectively added in the supernatant (2 μg/per portion) and incubated at 4 °C overnight in rotating equipment. After centrifugation, the precipitates were collected and washed with cold lysis buffer for six times. The mixture of precipitates and loading buffer was boiled and then Ac-SREBP1c, p300, SREBP1c levels were detected using immunoblotting.

### Liver-conditional knockout (CKO) *Pes1* gene in mice

The parent of *Pes1* CKO mice were generated by collaboration with Nanjing Institute of Biomedicine, Nanjing University (Jiangsu, China). Positive F1 generation mice were achieved by CRISPR–Cas9 technology. And hepatic *Pes1* CKO mice were obtained by positive F1 generation crossing with ALB-Cre mice. Genotyping was performed by PCR using genomic DNA extracted from murine tails at 3–4 weeks. The mice were classified into wild-type littermates (fl/fl, wt/wt) and *Pes1*^(−/−)^ (fl/fl, mut/wt) relying on genotype. The primer sequences for the transgenic Cre mice were as follows: Cre sense, 5ʹ-TTGGCCCCTTACCATAACTG-3ʹ; Cre antisense, 5ʹ-GAAGCAGAAG CTTAGGAAGATGG-3ʹ. The primer sequences for genotyping the *Pes1* alleles were as follows: shared sense, 5ʹ-TTCCTCACCCTCAGCATTAGʹ; wild-type antisense, 5ʹ-GAGATAGACTGCAAGGCACTGT-3ʹ. Only male mice were used for all analyses (10–15 mice per group). After 16 weeks, mice were sacrificed after anesthetization to obtain the serum and livers for consecutive measuring TG, TC, and running Western Blotting and qRT-PCR.

### Statistical analysis

Normally distributed data were expressed as mean ± SEMs. The continuous variables were analyzed either by Student’s t test, or by ANOVA followed by Student–Newman–Keuls q test for multiple comparisons. *P* < 0.05 was considered statistically significant. The data were analyzed using SPSS version 22.0 (IBM Corporation, Armonk, USA). Graphics were constructed using GraphPad Prism 7 (GraphPad Software, San Diego, CA).

## Results

### Long-term KD intervention improved the hyperglycemia and insulin resistance in diabetic mice

**Fig. 1:**
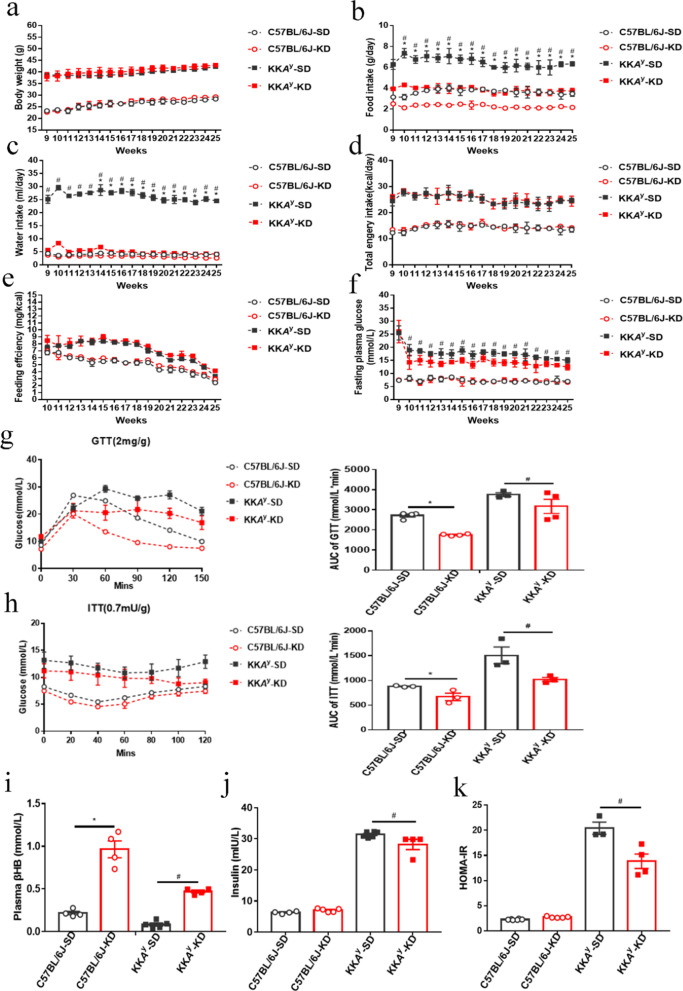
Ketogenic diet decreased the hyperglycemia and ameliorated insulin resistance in diabetic mice. **a** Shown are the changes of body weights exerted by the different food feedings in normal and diabetic mice. **b** Displayed are the food intakes in different groups throughout 16 weeks of feeding. **c** Exhibited are the water intakes in different groups throughout 16 weeks. **d** Demonstrated are the total energy intakes calculated by calories in different groups throughout 16 weeks. **e** Unveiled are the feeding efficiencies in different groups (calculated as body weight gain (mg) per kcal food consumed). **f** Shown are the variations of fasting plasma glucose in different groups throughout 16 weeks. **g** Intraperitoneal glucose tolerance tests were performed in different groups by the end of food feeding. **h** Intraperitoneal insulin tolerance tests were carried out in different groups by the end of food feeding. Glucose (2 mg/g) or insulin (0.75 mU/g) was injected intraperitoneally after mice were fasted for 6–8 h. Blood glucose samples were collected at different indicated time points and measured. Areas under the curve were calculated for the quantitative analysis. **i** Demonstrated are the levels of plasma β-hydroxybutyric acid (β-HB) in different groups. **j** Insulin levels were detected in different groups by the end of food feeding. **k** HOMA-IR in different groups was calculated by the formula. Data were shown as mean ± SEM for each experiment performed independently 3 times (n = 10–12 per group). SD (Standard diet), KD (Ketogenic diet). *P < 0.05 C57BL/6J-KD vs C57BL/6J-SD, ^#^P < 0.05 KK*A*^y^-KD vs KK*A*^y^-SD (ANOVA, Student–Newman–Keuls q test)

After 16 weeks of KD intervention, murine body weights in KK*A*^y^-KD group showed no significant difference from those in the KK*A*^y^-SD groups, the similar results were also observed in the C57BL6J mice (Fig. 1a). With regard to both normal and diabetic mice, intakes of SD were significantly higher than those of KD (Fig. 1b), parallel to the consumptions of water (Fig. 1c). In terms of daily energy intake and feeding efficiency, the values by the KD and SD feeding in normal and diabetic mice were not drastically different (Fig. 1d, e). However, the fasting plasma glucose levels were remarkably reduced in diabetic mice by KD intervention, compared to those by SD (Fig. 1f).

The ITT and GTT assays exhibited that KD could significantly ameliorate glucose tolerance and insulin sensitivity in diabetic mice (Fig. 1g, h). Moreover, serum insulin levels and HOMA-IR in diabetic mice were obviously improved by KD treatments (Fig. 1j, k), but no statistic difference between C57BL/6J-SD and C57BL/6J-KD mice was observed. In addition, the serum β-HB levels were sharply elevated by KD feeding in both healthy and diabetic mice (Fig. 1i).

### KD suppressed PES1 and improved lipid dysregulation under diabetic state

**Fig. 2:**
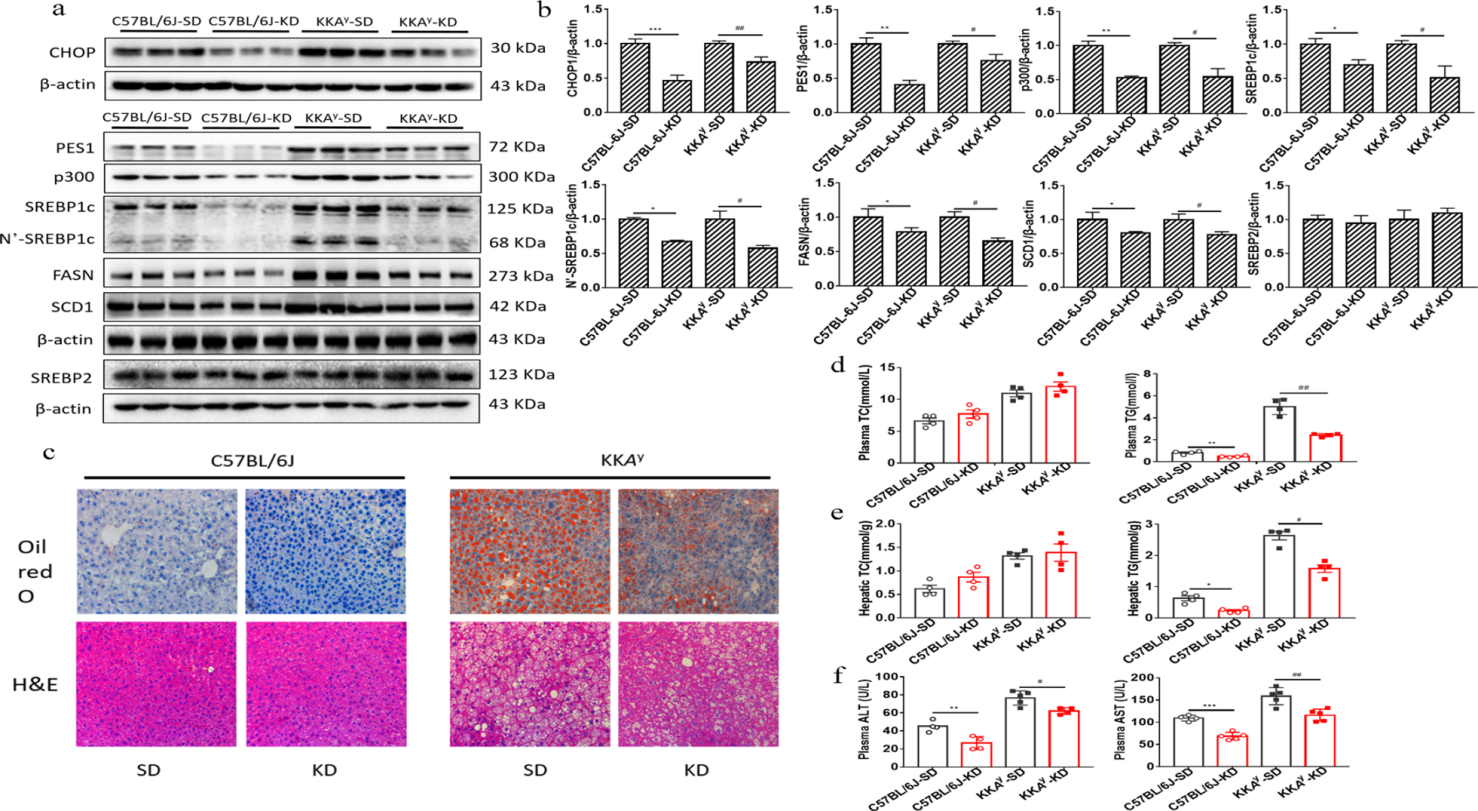
KD suppressed the PES1 expression and decreased plasma and hepatic TG levels in normal and diabetic mice. **a**, **b** The protein levels of hepatic CHOP, PES1, p300, SREBP1c, SREBP2, FASN and SCD1 were detected by Immunoblotting. **c** The oil red O and H&E staining of liver tissues were performed for different groups, original magnification, ×10. Scale bar, 50 μm. **d** Plasma TG and TC were assayed for different groups by the end of food feeding. **e** Hepatic TG and TC levels were detected for different groups. **f** Plasma AST and ALT levels were measured by Reagent kit. Values are means ± SEM for each experiment performed independently 3 times. SD (Standard diet), KD (Ketogenic diet). *P < 0.05, **P < 0.01, ***P < 0.001 compared with control for C57BL/6J, ^#^P < 0.05, ^##^P < 0.01, ^###^P < 0.001 compared with control for KK*A*^y^ (ANOVA, Student–Newman–Keuls q test)

After 16 weeks of KD feeding, the current results displayed that the protein levels of CHOP, PES1, p300, SREBP1c, N’-SREBP1c, FASN and SCD1 were dramatically inhibited in both healthy and diabetic mice (Fig. 2a, b). Histological analysis (H&E and Oil Red O staining) revealed much less pathogenesis and lipid accumulation in livers of KK*A*^y^ mice fed by KD than those by SD (Fig. 2c). Additionally, KD extraordinarily decreased plasma and hepatic TG levels in C57BL/6J and KK*A*^y^ mice, compared with SD (Fig. 2d, e). Lastly, KD feeding in normal and diabetic mice was associated with reductions of plasma AST and ALT (Fig. 2f).

### β-HB decreased PES1 and triglycerides in liver cells

**Fig. 3:**
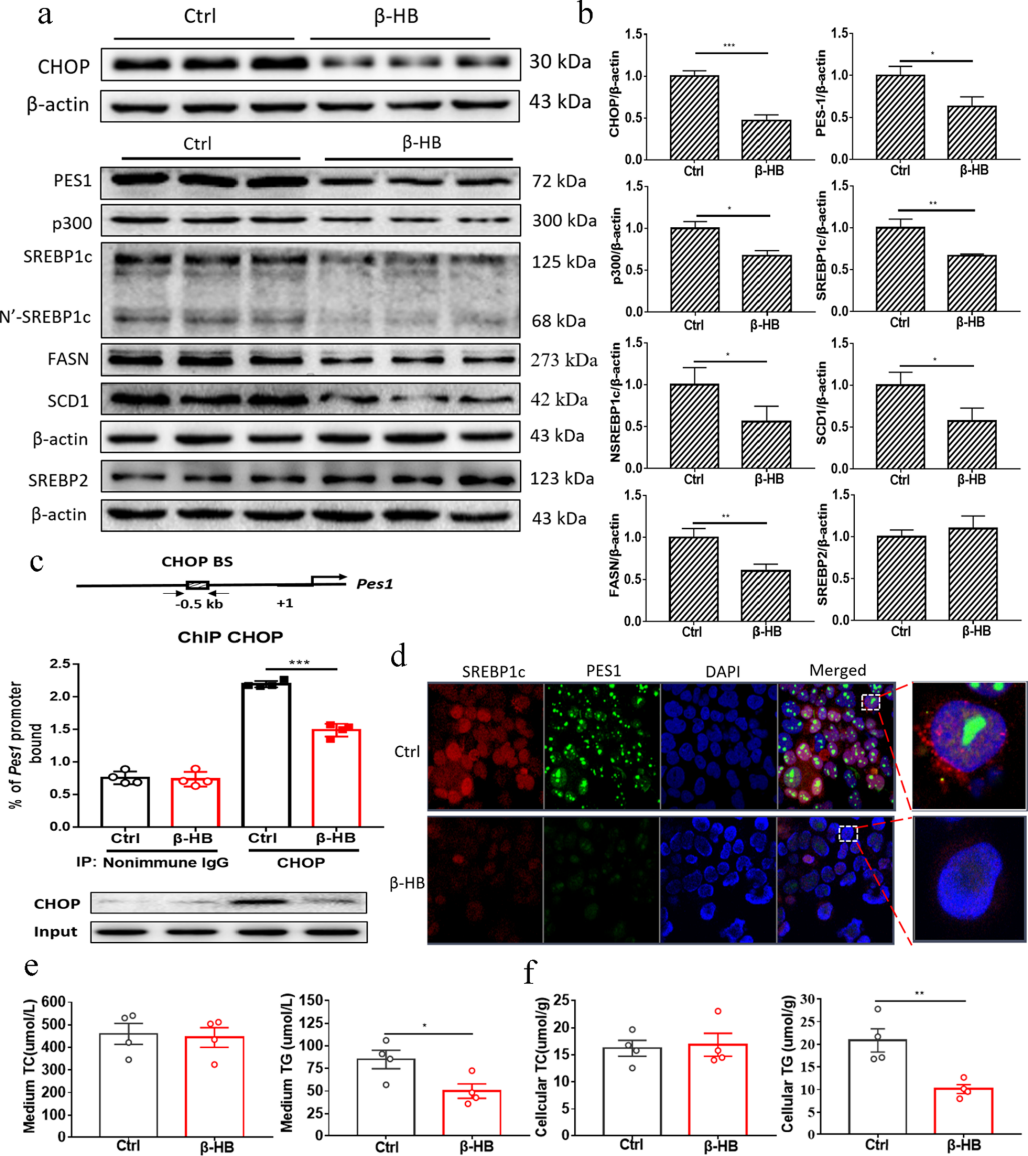
β-HB decreased PES1 and triglycerides in cultured liver cells. **a**, **b** The protein levels of CHOP, PES1, p300, SREBP1c, SREBP2, FASN and SCD1 in hepatocytes were detected by immunoblotting after 1 mM β-HB treatment for 24 h. **c** β-HB decreased CHOP binding to *Pes1* promoter. **d** Exhibited are immunofluorescence images of β-HB-treated liver cells for PES1 and SREBP1c expression and localization, Scale bar represents 20 um. The nuclei were stained with DAPI. **e**, **f** Demonstrated are the TG and TC levels respectively detected in the cultured media and liver cells after β-HB treatment. Ctrl (Control), β-HB (β-hydroxybutyric acid). Data were shown as mean ± SEM for each experiment performed independently 3 times. *P < 0.05, **P < 0.01, ***P < 0.001 compared with control (Student’s t test)

β-HB, the main component of ketone bodies, was used to test the KD effect on liver cells in vitro. The optimized concentration and time of β-HB treatment were selected by cell viability test (Additional file 1: Fig. S1a). The protein levels of PES1 by the β-HB treatments with different concentrations and times were dramatically lower than those by control treatment (Additional file 1: Fig. S1b, c). Considering the ketogenesis by the KD feeding in mice, the concentration (1 mM) of β-HB and its treatment time (24 h) were identified for liver cell treatment in vitro, by which the protein levels of CHOP, PES1, p300, SREBP1c, N’-SREBP1c, FASN and SCD1 were greatly attenuated (Fig. 3a, b). In addition, ChIP test indicated that β-HB decreased CHOP binding to *Pes1* promoter (Fig. 3c). Consistent with the above data, the immunofluorescence showed that β-HB suppressed PES1 and SREBP1c proteins in nucleus of McArdle cells (Fig. 3d). Moreover, medium and cellular TG levels were markedly declined by β-HB treatment (Fig. 3e, f).

### In vitro knockdown of *Pes1* impaired medium and cellular triglyceride production in liver cells

**Fig. 4:**
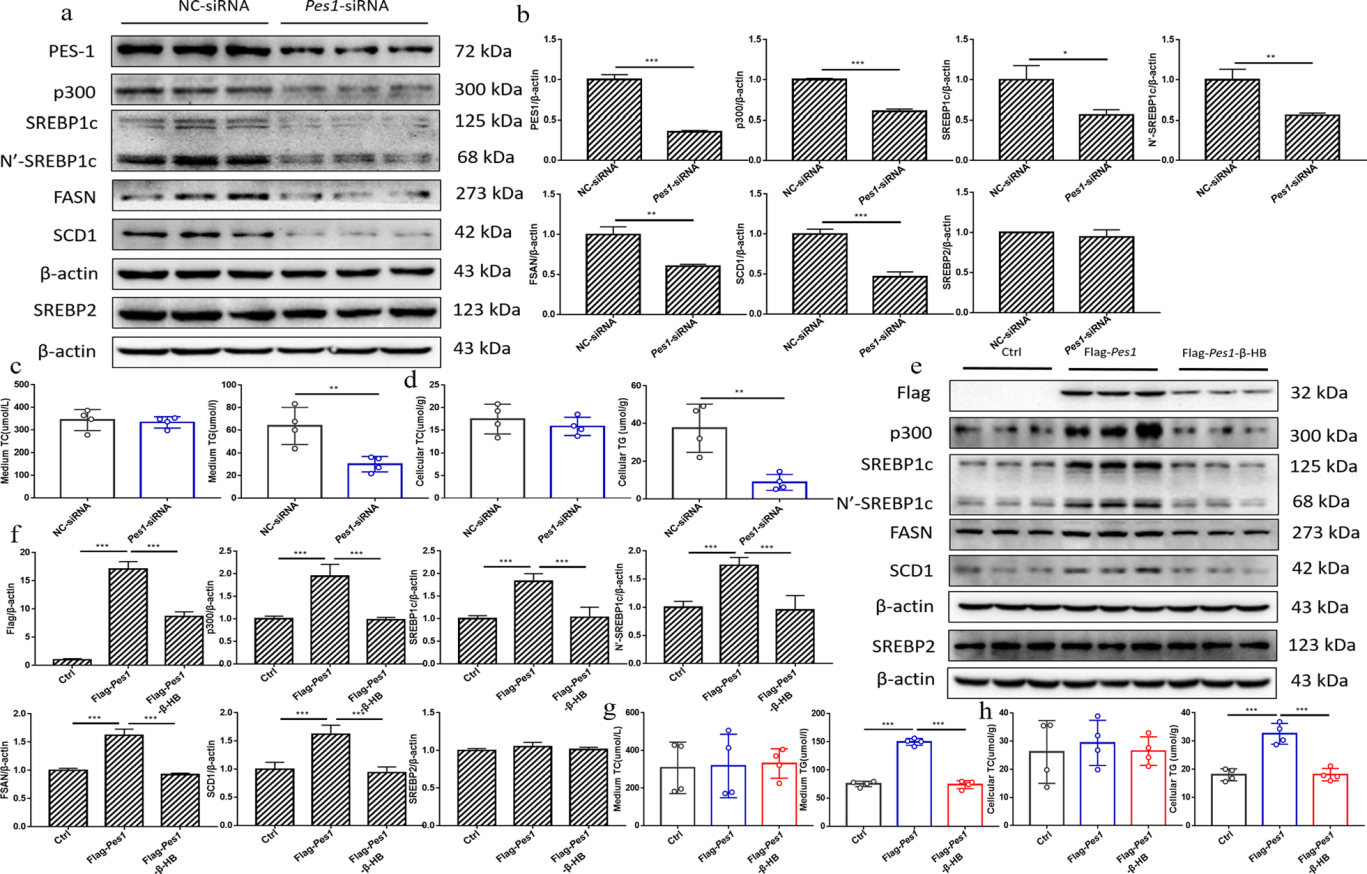
Effects of *Pes1* knockdown and overexpression on lipid metabolism in vitro. **a**, **b** *Pes1* knockdown in cultured liver cells inhibited the protein levels of PES1, p300, SREBP1c, FASN and SCD1. **c**, **d** Displayed are the effects of *Pes1* knockdown on the TG and TC levels in cultured liver cells and media. **e**, **f** *Pes1* overexpression enhanced PES1-Flag, SREBP1c, p300, FASN and SCD1 protein levels in cultured liver cells, while β-HB treatment abolished the effects. **g**,** h** Exhibited are the effects of *Pes1* overexpression on the TG and TC levels in cultured liver cells and media. Data were shown as mean ± SEM for each experiment performed independently 3 times. *P < 0.05, **P < 0.01, ***P < 0.001 compared with controls (Student’s t-test or ANOVA, Student–Newman–Keuls q test)

To probe PES1 modulating role, the siRNA knockdown of *Pes1* was performed in McArdle cells. The levels of PES1 protein were significantly downregulated in the treated cells (Fig. 4a, b). Simultaneously, the medium and cellular triglycerides, and the levels of p300, SREBP1c, N’-SREBP1c, FASN and SCD1 in the treated cells were dramatically reduced compared with those in control cells (Fig. 4a–d).

### In vitro supplementation of *Pes1* promoted cellular triglyceride levels, but β-HB treatment eliminated the elevation

The overexpression of *Pes1 *in vitro was performed to further confirm the key role of PES1 in lipid metabolism. The current results showed that the protein levels of p300, SREBP1c, N’-SREBP1c, FASN and SCD1 were significantly increased by *Pes1* overexpression, but the effects were sharply reversed by β-HB treatment (Fig. 4e, f). Furthermore, overexpression of *Pes1* significantly increased medium and cellular triglycerides, while β-HB treatment eliminated those increases (Fig. 4g, h).

### Liver-specific *Pes1* knockout in mice decreased the lipid generation

**Fig. 5:**
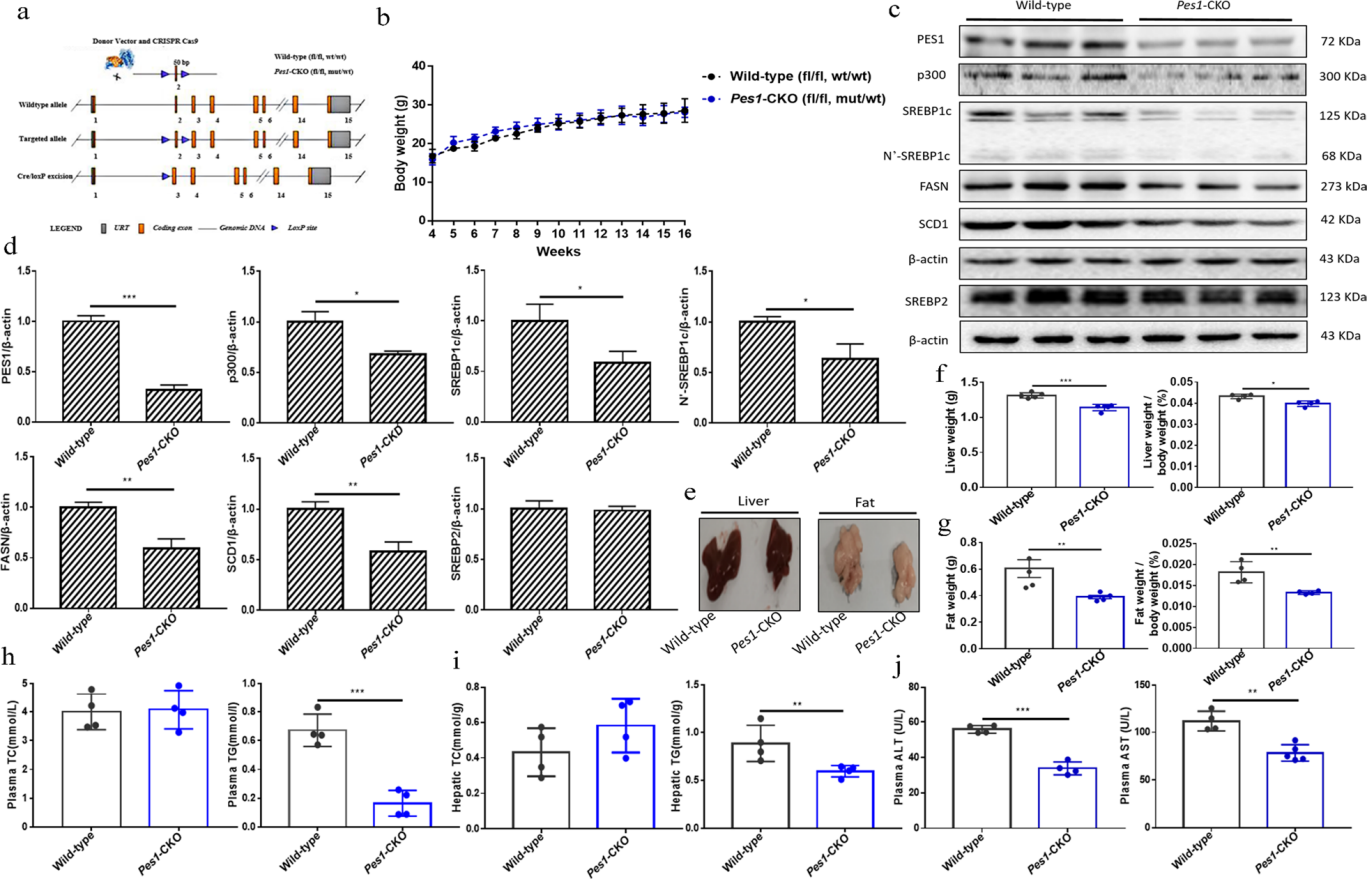
Effects of liver-specific *Pes1* knockout on lipid metabolism in vivo. **a** Displayed is the scheme of *Pes1* gene knockout strategy. **b** Shown are the effects of *Pes1* knockout on the murine body weights. **c**, **d** *Pes1* knockout in mice eliminated the protein levels of PES1, and inhibited p300, SREBP1c, FASN and SCD1 proteins. **e**–**g** Shown are the effects of *Pes1* knockout on the weights of livers and fat. **h**, **i** Demonstrated are the effects of *Pes1* knockout on the TG and TC levels in murine plasma and livers. **j** Plasma AST and ALT levels were measured by Reagent kit. Data were shown as mean ± SEM for each experiment performed independently 3 times. *P < 0.05, **P < 0.01, ***P < 0.001 compared with control (Student’s t test)

The exon 2 in murine *Pes1* gene was targeted by CRISPR–Cas9/RNA system gene targeting technology (Fig. 5a). The liver mRNA levels of *Pes1* were almost completely eliminated in CKO mice by qRT-PCR test (Additional file 1: Fig. S2). After 16 weeks of feeding, no significant difference of body weights between wild-type littermates (fl/fl, wt/wt) and *Pes1*-CKO mice (fl/fl, mut/wt) was found (Fig. 5b). But the protein levels of p300, SREBP1c, N’-SREBP1c, FASN and SCD1 were dramatically downregulated in *Pes1*-CKO mice (Fig. 5c, d). Moreover, the weights of liver and subcutaneous fat, and the ratio of liver or fat to body weight were also substantially declined in *Pes1*-CKO mice (Fig. 5e–g). Likewise, plasma and hepatic triglycerides, plasma AST and ALT in *Pe*s1-CKO mice were sharply lower than those in the wild-type littermates (Fig. 5h–j).

### β-HB lowered PES1 binding to the *p300* promoter and downregulated PES1 mediated acetylation of SREBP1c

**Fig. 6:**
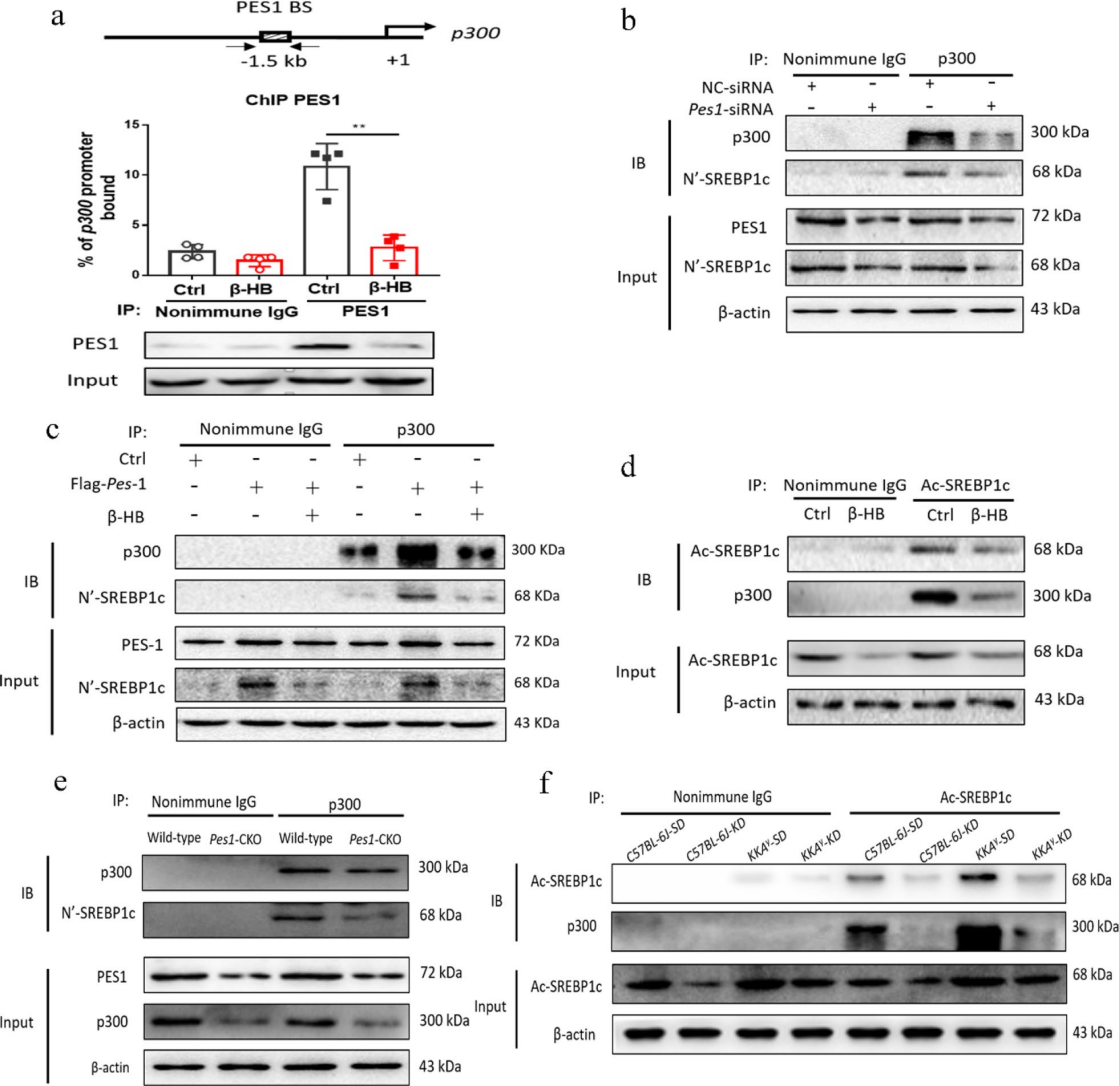
β-HB reduced PES1 binding to the *p300* promoter and downregulated PES-1 mediated acetylation of SREBP1c. **a** β-HB decreased PES1 binding to the *p300* promoter. **b**, **c** PES1 knockdown or overexpression inhibited or enhanced the interaction between p300 and SREBP1c in hepatocytes. **d** β-HB treatment decreased the acetylation of SREBP1c in hepatocytes. **e** *Pes1* knockout impaired the interaction between p300 and SREBP1c in mice. **f** KD feeding decreased the acetylation of SREBP1c in normal and diabetic mice. Data were shown as mean ± SEM for each experiment performed independently 3 times. *P < 0.05, **P < 0.01, ***P < 0.001 compared with control (ANOVA, Student–Newman–Keuls q test)

The ChIP experiment revealed that β-HB reduced PES1 binding to the *p300* promoter, resulting in downregulation of *p300* gene expression (Fig. 6a). To determine whether β-HB affects PES1 regulated SREBP1c activity, acetylation assays both in vitro and in vivo were performed. Additionally, co-IP was used to explore the interaction between p300 and SREBP1c. We found that *Pes1* silence in hepatocytes reduced the interaction between p300 and SREBP1c (Fig. 6b). Contrastingly, *Pes1* overexpression increased the interaction, which was however impaired under β-HB treatment (Fig. 6c). Subsequently, β-HB treatment in hepatocytes inhibited the acetylation of SREBP1c (Fig. 6d). Next, whether the acetylation of SREBP1c is regulated by PES1 in vivo was also investigated. Our current data indicated that *Pes1* knockout in mice significantly reduced the interaction between p300 and SREBP1c (Fig. 6e). And KD feeding in normal and diabetic mice showed that the acetylation of SREBP1c was sharply suppressed (Fig. 6f).

### KD and β-HB decreased inflammation responses by downregulating PES1

**Fig. 7:**
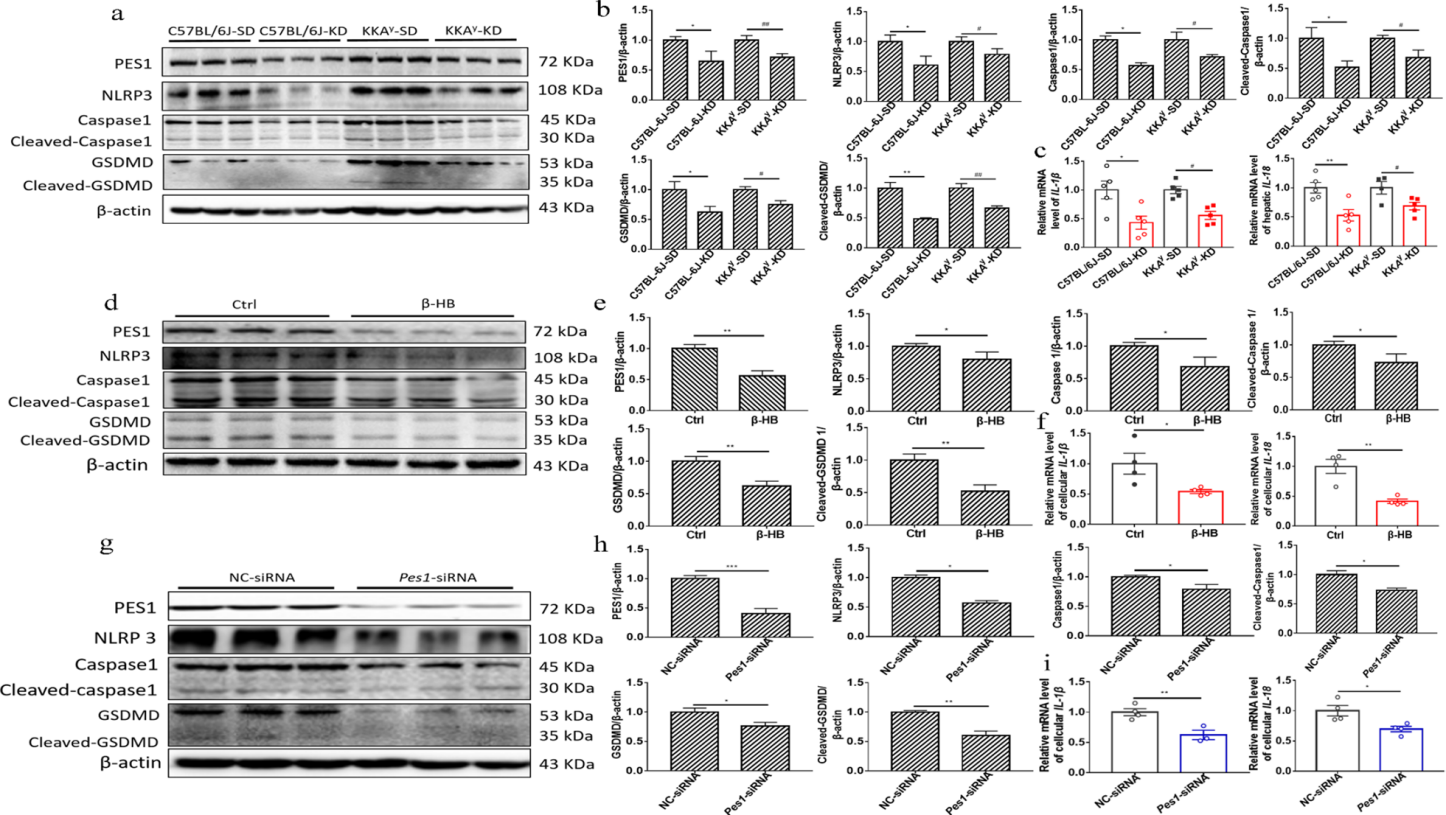

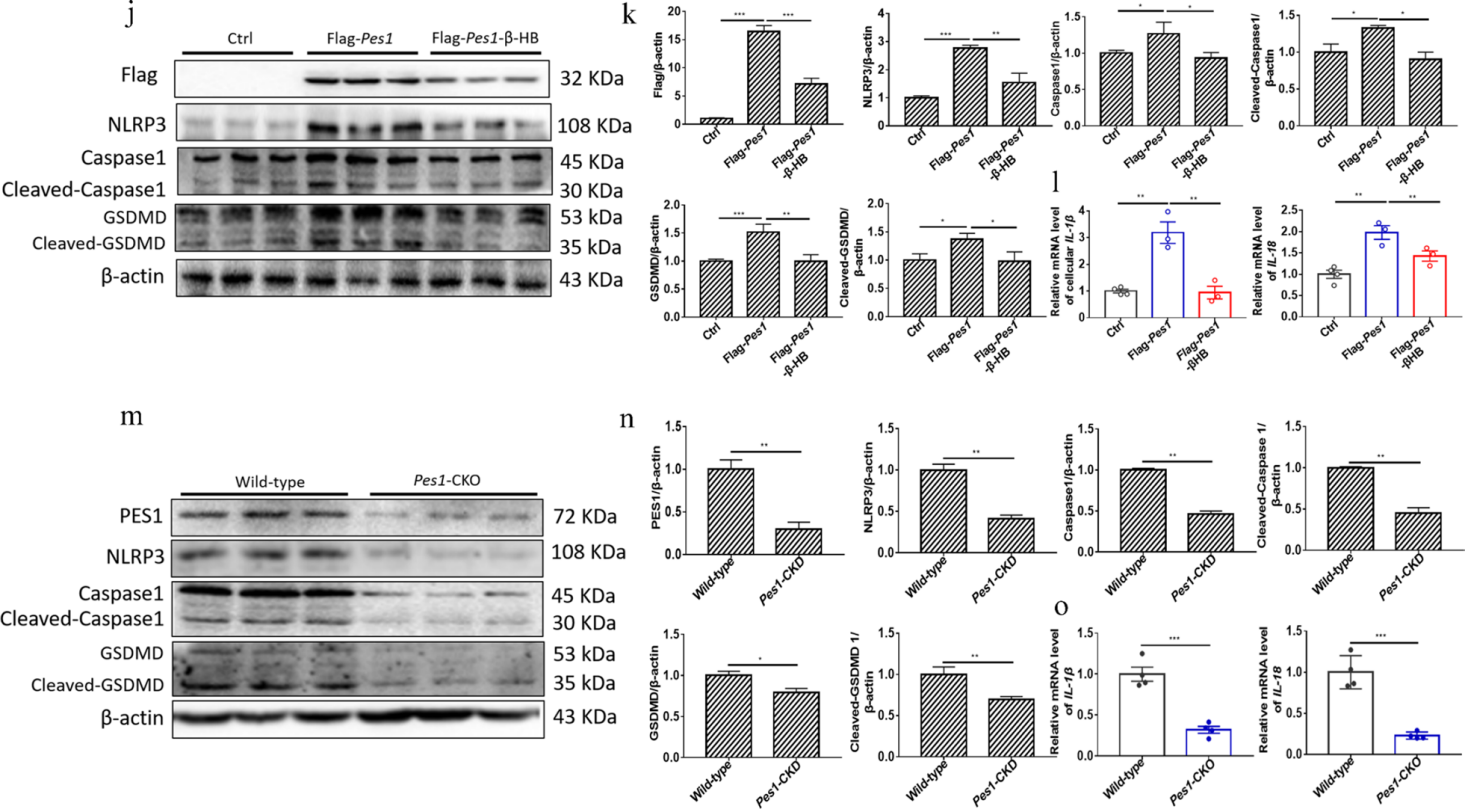
KD feeding and β-HB treatment decreased inflammation responses by downregulating PES1. **a**, **b** KD feeding in normal and diabetic mice suppressed the inflammation related proteins. **c** Relative mRNA levels of hepatic *IL-1β* and *IL-18* were measured by qRT-PCR. **d**, **e** β-HB treatment in hepatocytes inhibited the inflammation proteins. **f** Relative mRNA levels of *IL-1β* and *IL-18* in cultured hepatocytes were measured by qRT-PCR. **g**, **h** *Pes1* knockdown in hepatocytes lowered the inflammation proteins. **i** Shown are the effects of *Pes1* knockdown on the relative mRNA levels of cellular *IL-1β* and *IL-18*. **j**, **k** *Pes1* supplementation in hepatocytes enhanced the levels of inflammation proteins. **l** Exhibited are the effects of *Pes1* supplementation on the relative mRNA levels of cellular *IL-1β* and *IL-18*. **m**, **n** *Pes1*-CKO in mice reduced inflammation proteins in murine livers. **o** Demonstrated are the effects of *Pes1* knockout on the relative mRNA levels of hepatic *IL-1β* and *IL-18*. Data were shown as mean ± SEM for each experiment performed independently 3 times. *P < 0.05, **P < 0.01, ***P < 0.001 compared with controls or ^#^P < 0.05, ^##^P < 0.01, ^###^P < 0.001 compared with controls (Student’s t-test or ANOVA, Student–Newman–Keuls q test)

According to the published reports, inflammation might promote lipogenesis (Todoric et al. 2020). In the present study, the levels of Recombinant NLR Family Pyrin Domain Containing Protein 3 (NLRP3), Caspase1, Cleaved-Caspase1, gasdermin-D (GSDMD), and Cleaved-GSDMD were found to be significantly inhibited in normal and diabetic mice fed with KD, compared with those fed with SD (Fig. 7a, b). Simultaneously, KD led to much less inflammation responses in mice than did SD, as indicated by decreased pro-inflammatory factor levels (*IL-1β* and *IL-18*) in the murine livers (Fig. 7c), similar to the results of β*-*HB treated hepatocytes (Fig. 7d–f). Furthermore, the silence or supplementation of *Pes1 *in vitro elicited the suppression or enhancement of NLRP3, Caspase 1, Cleaved-Caspase 1, GSDMD, Cleaved-GSDMD, *IL-1β* and *IL-18* levels, respectively (Fig. 7g–i). More interestingly, the enhancement by overexpression of *Pes1* was also impaired with β*-*HB treatment in vitro (Fig. 7j–l). In addition, consistent with *Pes1* silence in vitro, the similar results were observed in *Pes1* knockout mice (Fig. 7m–o).

### β-HB decreased PES1 binding to the *Caspase1* promoter and knockdown of Caspase1 reduced medium and cellular triglycerides in liver cells

**Fig. 8:**
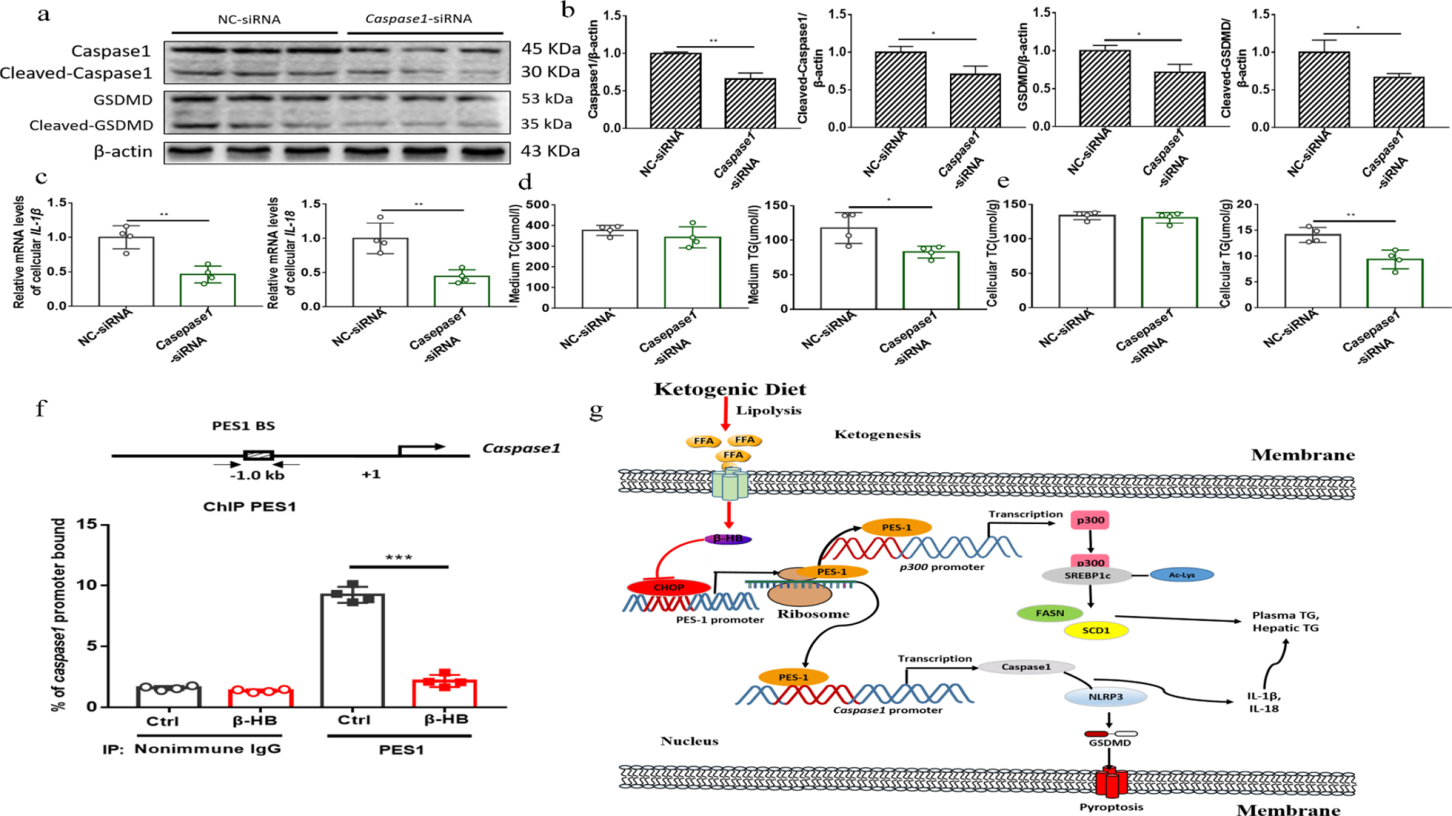
β-HB lowered PES1 binding to the *Caspase1* promoter and *Caspase1* knockdown in hepatocytes reduced the medium and cellular TG. **a**, **b** *Caspase1* knockdown in liver cells suppressed the inflammation proteins. **c** Shown are the effects of *Caspase1* knockdown on the relative mRNA levels of cellular *IL-1β* and *IL-18*. **d**, **e** *Caspase1* knockdown in liver cells reduced the medium and cellular TG. **f** β-HB decreased PES1 binding to the *Caspase1* gene promoter. **g** Schematic diagram of the entire study. Data were shown as mean ± SEM for each experiment performed independently 3 times. *P < 0.05, **P < 0.01, ***P < 0.001 compared with control (Student’s t test)

To verify Caspase1 modulating role, the siRNA knockdown of *Caspase1* was carried out in hepatocytes. The knockdown results suggested that the levels of Caspase1 and GSDMD were declined (Fig. 8a, b), and that the mRNA levels of *IL-1β* and *IL-18* were suppressed as well (Fig. 8c). Furthermore, medium and cellular TG levels were dramatically inhibited in *Caspase1* siRNA treated cells (Fig. 8d, e). Mechanistically, β-HB treatment in liver cells lowered PES1 binding to *Caspase1* gene promoter, resulting in the downregulation of *Caspase1* gene expression (Fig. 8f). The schematic diagram of entire study is presented in the Fig. 8g.

## Discussion

Recently, clinical studies indicated that KD was effective in improving metabolic parameters of weight, glycemia, and lipid profiles in patients with overweight or obesity, especially in those with preexisting diabetes (Choi et al. 2020; Li and Heber 2020). In our current study, although the high glucose level is naturally dropped in KK*A*^y^ murine model after age of 18 weeks according to the literature (Zhang et al. 2020), we still observed the effects of KD on lowering plasma glucose and improving insulin sensitivity in T2DM mice compared with those of SD, which was consistent with previous publication (Yang et al. 2021). In addition, Guo et al. (2020) also reported that the plasma and hepatic TG levels were evidently dropped in normal and T2DM mice fed by KD, which are exactly resembling to our current study.

β-HB, as the predominant component of ketone bodies, has been demonstrated to serve as molecular scaffold that regulates cellular function by directly activating hydroxy-carboxylic acid receptors, and to be involved in multiple physiological processes including metabolism and inflammation (Offermanns 2017. In this study, the levels of β-HB in normal mice were higher than those in diabetic mice after KD treatment. This may be because the ketone bodies are produced by fatty acid β-oxidation, which is upregulated by carnitine palmitoyltransferase 1 (CPT1) (Puchalska and Crawford 2017). Similarly, 3-hydroxymethylglutaryl-CoA synthase 2 (HMGCS2) is a rate-limiting enzyme that promotes β-HB synthesis as well (Shimazu et al. 2010). In our current work, although the expression of CPT1 and HMGCS2 by KD treatment is higher than that by SD in both C57BL/6J and KK*A*^y^ mice, the latter ones fed with SD or KD had much less expression of CPT1 and HMGCS2 than the previous ones (Additional file 1: Fig. S3). These results suggested that the β-HB levels in KK*A*^y^ mice may be lower than those in C57BL/6J mice after 16-week treatment with the same KD, as shown in our Fig. 1i. In addition, emerging evidence from long-term KD intervention in humans supports that the ketone level was also attenuated in T2DM patients (Landry et al. 2021). And the initial β-HB value was below ketosis level at the beginning of the KD treatment (0.12 mmol/L), then peaked at 1.1 mmol/L (4 weeks) and decreased to 0.51 mmol/L by week 12 in the end (Landry et al. 2021). Moreover, β-HB levels are significantly different among different mice by KD intervention based on previous reports. For example, the β-HB levels in *db/db* mice were dramatically higher than those in wild type mice after KD intervention, while the enhanced degree of β-HB levels in *ob/ob* mice relative to their controls fed with the same KD were much lower than that in *db/db* mice (Thio et al. 2006; Poplawski et al. 2011; Badman et al. 2009). Furthermore, our current data indicated that the elevation fold of β-HB level in KK*A*^y^ mice was even moderately higher than that in C57BL/6J mice comparing KD feeding to SD, but no significant difference between two ratios was observed (Additional file 1: Fig. S4). Additionally, the fasting plasma glucose, HOMA-IR and TG levels were markedly reduced in KD-fed KK*A*^y^ mice compared with SD-fed controls, suggesting the effective intervention by KD-derived β-HB levels in vivo. Taken together, although the ketogenesis by KD was seemingly mitigated in KK*A*^y^ mice compared with that in C57BL/6J mice, the ketone level was still legitimate for effectiveness in KK*A*^y^ mice in our current study based on the preceding findings and present results.

Of note, we discerned that PES1 levels were downregulated by KD feeding in normal and diabetic mice and by β-HB treatment in cells. Previous finding indicated that circANRIL could play an important role in atheroprotection by inhibiting PES1 (Holdt et al. 2016). Moreover, our current study unveiled that β-HB may reduce CHOP binding to *Pes1* promoter, resulting in the downregulation of PES1. The decreased PES1 protein level impaired its binding to *p300* and *Caspase1* promoters, causing the declined expressions of p300 and Caspase1, thus undermining p300 mediated acetylation of SREBP1c and inflammation response. CHOP, a key apoptosis-promoting factor, tightly controls the gene transcription and has pivotal roles in lipid accumulation, adipogenesis and inflammatory action (Liu et al. 2018; Maris et al. 2012; Allagnat et al. 2012). And one report has indicated that KD intervention in mice could reduce the expression of CHOP (Yu et al. 2020), which is consistent to our current study. In addition, it has also been proposed that SREBP1c binds to its lipogenic target genes, such as fatty acid synthase (FASN) and stearoyl-CoA desaturase 1 (SCD1), thereby stimulating de novo lipogenesis (Li et al. 2015). A crucial role of SREBP1c has been shown in vivo by hepatic overexpression of SREBP1c in transgenic mice that led to increased hepatic lipid accumulation (Wang et al. 2015). Accumulating evidence showed that the acetylation of SREBP1c increased its stability and activity, and SREBP1c is acetylated by p300 at Lys-289 and Lys-309 (Ponugoti et al. 2010). In this report, after *Pes1* gene was knocked down, the interaction between SREBP1c and p300 was significantly inhibited, consistent with our observation in *Pes1*-CKO mice. Moreover, the interaction between SREBP1c and p300 was obviously strengthened during *Pes1* gene overexpression, which was however impeded by β-HB treatment. Furthermore, our current study also suggested that β-HB and KD remarkably reduced the SREBP1c activity via inhibiting the acetylation of SREBP1c.

Compelling evidence based on animal study suggested that KD may block NLRP3 inflammasome-mediated IL-1β and IL-18 production (Youm et al. 2015), which is in concordance with our current observation. In addition, after in vitro knockdown of *Pes1* and in vivo ablation of *Pes1*, the expression of NLRP3, Caspase1 and GSDMD was significantly inhibited. This is opposite to that after overexpression of *Pes1 *in vitro. However, the *Pes1* overexpression induced effect was abolished by β-HB treatment. These results suggested that β-HB plays a vital role in regulation of inflammation response by downregulating PES1. Moreover, Caspase1 and NLRP3, as regulatory molecules of lipid metabolism, were reported to promote the release of mature IL-1β and to interfere insulin signaling and production, subsequently leading to the lipogenesis and fat accumulation (Anand 2020). Based on *Caspase1* knockdown in hepatocytes, our current data exhibit that medium and cellular TG levels are clearly curbed, which is similar to the protection role in *Caspase1* knockout mice fed with high fat diet (Stienstra et al. 2011). Collectively, these results suggest that both β-HB treatment in cells and KD feeding in mice greatly reduced lipid production or accumulation via downregulating PES1 modulated acetylation of SREBP1c and inflammation pathways.

The current study explored a novel mechanism of KD intervention in diabetic mice, which may have translational potential and clinical implications by finding a PES1 inhibitor. However, the interpretation of this study so far is still limited due to the lack of human intervention data, and PES1 function and its mediated mechanism by KD intervention in human is needed in future. In addition, the metabolic factors regulating PES1 expression under T2DM condition are not elucidated yet.

## Conclusion

In summary, our current findings demonstrate that KD may ameliorate lipid deposition in type 2 diabetic mice by downregulating PES1 modulated acetylation of SREBP1c and inflammation pathways. This study may provide a novel insight to understanding the mechanisms underlying T2DM related lipid dysregulation and contribute a new pharmaceutical target for T2DM treatment.

## Supplementary Information

**Additional file 1: Figure S1.** Determination of cell viability. a Optimal time and concentration of β-HB treatment was determined based on the cell viability by CCK8 test, including concentration gradient (0, 0.25, 1, 2, 4 mM), treatment times (0, 12, 24, 48 h). b, c Protein levels of PES1 in hepatocytes were detected in different concentrations and times of β-HB treatment. Data were shown as mean ± SEM for each experiment performed independently 3 times. *P < 0.05, **P < 0.01, ***P < 0.001 compared with control (ANOVA, Student–Newman–Keuls q test). **Figure S2.** Hepatic *Pes1* gene transcription in CKO mice was detected by qRT-PCR. Data were shown as mean ± SEM for each experiment performed independently 3 times. *P < 0.05, **P < 0.01, ***P < 0.001 compared with control (Student’s t test). **Figure S3.** KD enhanced the fatty acid β-oxidation in normal and diabetic mice. a, b The protein levels of hepatic CPT1 and HMGCS2 were detected by Immunoblotting. Values are means ± SEM for each experiment performed independently 3 times. SD (Standard diet), KD (Ketogenic diet). *P < 0.05, **P < 0.01, ***P < 0.001 compared with control for C57BL/6J, ^#^P < 0.05, ^##^P < 0.01, ^###^P < 0.001 compared with control for KK*A*^y^, ^+^P < 0.05, ^++^P < 0.01, ^+++^P < 0.001 compared with C57BL/6J (ANOVA, Student–Newman–Keuls q test). **Figure S4.** The ratios of β-HB levels in the C57BL/6J and KK*A*^y^ mice fed with KD and SD. Values are means ± SEM for each experiment performed independently 3 times. SD (Standard diet), KD (Ketogenic diet).

## Abbreviations

ALT: Alanine aminotransferase
AST: Aspartate aminotransferase
AUC: Area-under curve
CCK-8: Cell counting kit-8
CHOP: C/EBP-homologous protein
CKO: Conditional knockout
ELISA: Enzyme linked immunosorbent assay
FASN: Fatty acid synthase
GTT: Glucose tolerance test
GSDMD: Gasdermin D
HOMA-IR: Homeostatic model assessment of insulin resistance
IR: Insulin resistance
ITT: Insulin tolerance test
KD: Ketogenic diet
NLRP3: Recombinant NLR Family Pyrin Domain Containing Protein 3
PBS: Phosphate-buffered saline
PES1: Pescadillo 1
qRT-PCR: Quantitative real-time PCR
SD: Standard diet
siRNA: Small interfering RNA
SREBP1c: Sterol regulatory element binding protein 1c
SREBP2: Sterol regulatory element binding protein 2
T2DM: Type 2 diabetes mellitus
TC: Total cholesterol
TG: Triglycerides
β-HB: β-Hydroxybutyric acid
