# Supplementary material for: Ketogenic diet ameliorates lipid dysregulation in type 2 diabetic mice by downregulating hepatic pescadillo 1
Source: Mol Med. 2022 Jan 3;28:1. doi: 10.1186/s10020-021-00429-6 (PMC8722053; doi:10.1186/s10020-021-00429-6)
Supplement: Supplementary file 1 — Additional file 1: Figure S1. Determination of cell viability. a Optimal time and concentration of β-HB treatment was determined based on the cell viability by CCK8 test, including concentration gradient (0, 0.25, 1, 2, 4 mM), treatment times (0, 12, 24, 48 h). b, c Protein levels of PES1 in hepatocytes were detected in different concentrations and times of β-HB treatment. Data were shown as mean ± SEM for each experiment performed independently 3 times. *P < 0.05, **P < 0.01, ***P < 0.001 compared with control (ANOVA, Student–Newman–Keuls q test). Figure S2. Hepatic Pes1 gene transcription in CKO mice was detected by qRT-PCR. Data were shown as mean ± SEM for each experiment performed independently 3 times. *P < 0.05, **P < 0.01, ***P < 0.001 compared with control (Student’s t test). Figure S3. KD enhanced the fatty acid β-oxidation in normal and diabetic mice. a, b The protein levels of hepatic CPT1 and HMGCS2 were detected by Immunoblotting. Values are means ± SEM for each experiment performed independently 3 times. SD (Standard diet), KD (Ketogenic diet). *P < 0.05, **P < 0.01, ***P < 0.001 compared with control for C57BL/6J, #P < 0.05, ##P < 0.01, ###P < 0.001 compared with control for KKAy, +P < 0.05, ++P < 0.01, +++P < 0.001 compared with C57BL/6J (ANOVA, Student–Newman–Keuls q test). Figure S4. The ratios of β-HB levels in the C57BL/6J and KKAy mice fed with KD and SD. Values are means ± SEM for each experiment performed independently 3 times. SD (Standard diet), KD (Ketogenic diet). [file 10020_2021_429_MOESM1_ESM.docx]

**Table S1** Primers for quantitative real-time-PCR

| Genes | Sequences | Sizes (bp) | Species |
| --- | --- | --- | --- |
| *β-actin* | Forward:5'-TTGTGTTGGACTCTGGTGATGGTG-3' | 81 | Rat |
|  | Reverse:5’-GACGGAGAATGGCATGTGGAAGG-3' |  |  |
| *IL-1β* | Forward:5'-ATGAGAGCATCCAGCTTCAA-3' | 90 | Rat |
|  | Reverse:5’-TGAAGGAAAAGAAGGTGCTC-3' |  |  |
| *IL-18* | Forward:5'-TCAAAGTGCCAGTGAACCCC-3' | 96 | Rat |
|  | Reverse:5’-GGTCACAGCCAGTCCTCTTAC-3' |  |  |
| *β-actin* | Forward:5'-TATGCTCTCCCTCACGCCATCC-3' | 129 | Mouse |
|  | Reverse:5’-GTCACGCACGATTTCCCTCTCAG-3' |  |  |
| *IL-1β* | Forward:5'-AAAATGCCTCGTGCTGTCTGA-3' | 125 | Mouse |
|  | Reverse:5’-CAGGGATTTTGTCGTTGCTTG-3' |  |  |
| *IL-18* | Forward:5'-CAGACCACTTTGGCAGACTTCA-3' | 127 | Mouse |
|  | Reverse:5’-ACACAGGCGGGTTTCTTTTGT-3' |  |  |

**Table S2** Ingredient composition of the diets

| Diet formula (g/1000g total diet) | SD | KD |
| --- | --- | --- |
| Casein | 178.0 | 83.0 |
| Corn starch | 396.4 | 83.0 |
| Maltodextrin | 194.0 | - |
| Sucrose | 52.6 | - |
| Soybean oil | 70.0 | - |
| Lard | - | 665.0 |
| Cellulose | 61.0 | 99.0 |
| Mineral Mix | 24.0 | 34.0 |
| Vitamin Mix | 20.0 | 32..2 |
| L-Cystine | 3.0 | 3.8 |
| Choline bitartrate | 1.0 | - |
| Total energy (kcal/g) | 3.8 | 6.6 |

Nutrient composition: standard diet: protein (17.8%), carbohydrate (64.3%), fat (7.0%); KD: protein (8.3%), carbohydrate (8.3%), fat (66.5%). SD, standard diet; KD, ketogenic diet.

**Fig. S1**

**
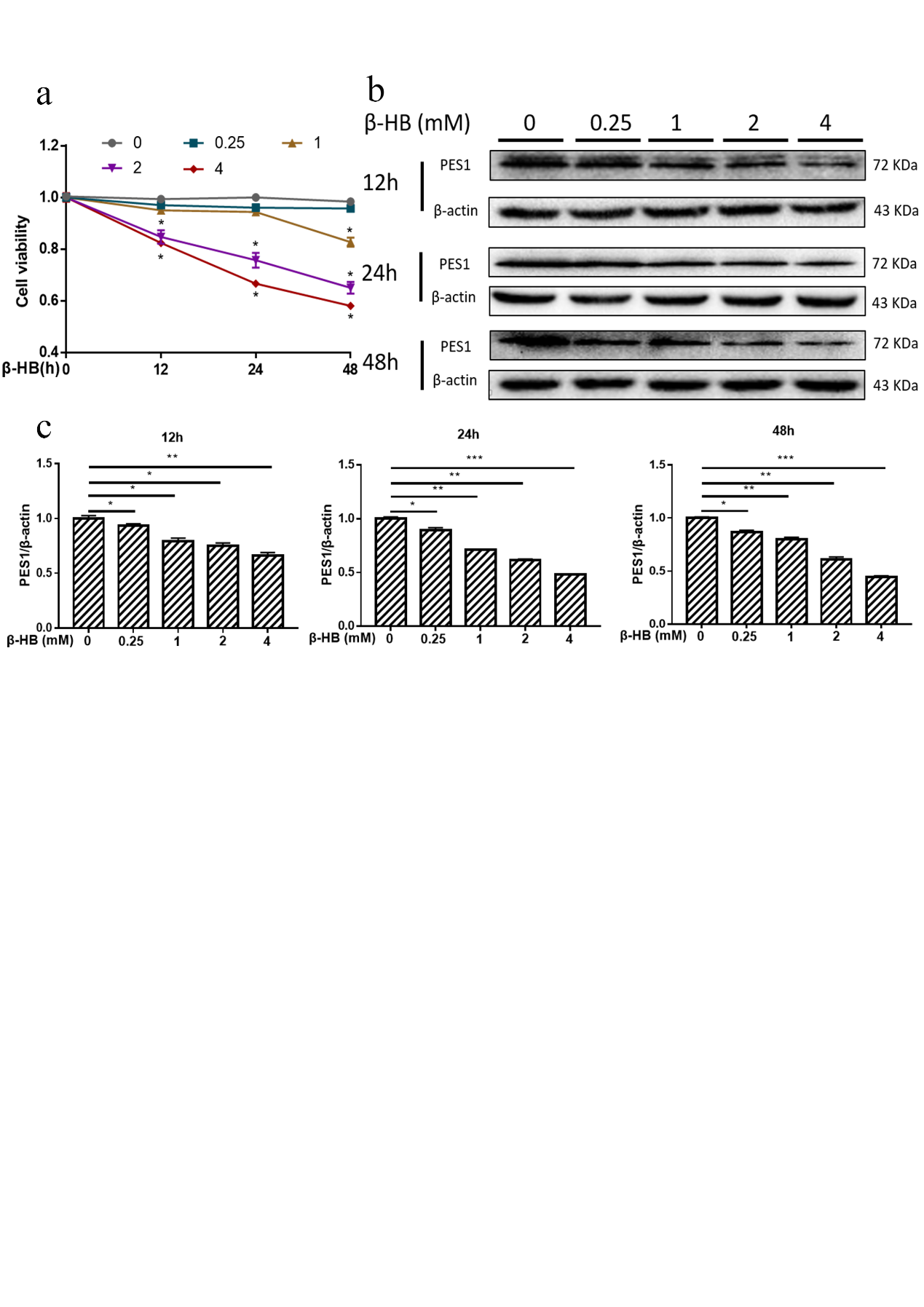
**

**Fig. S2**


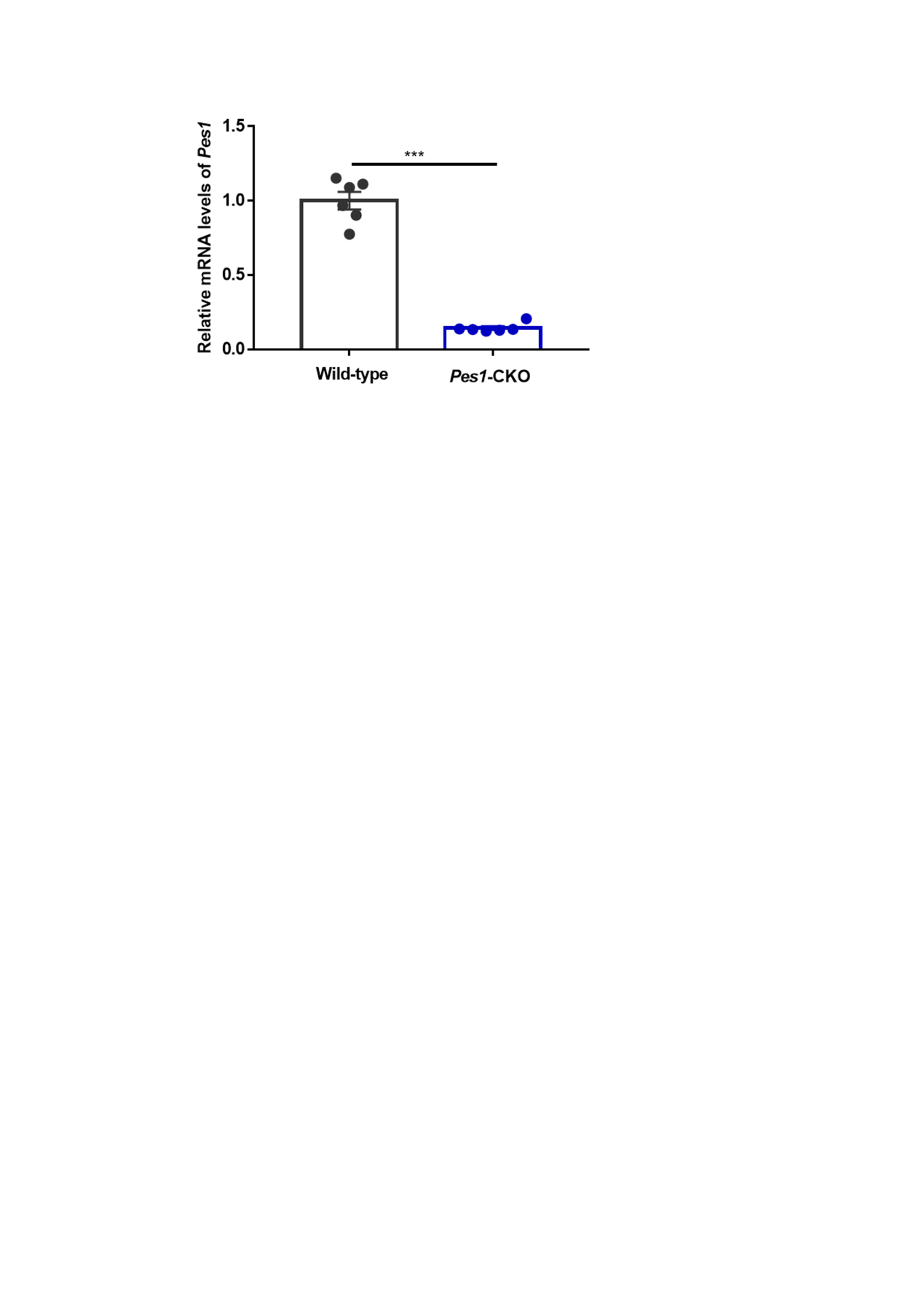


**Fig. S3**

*
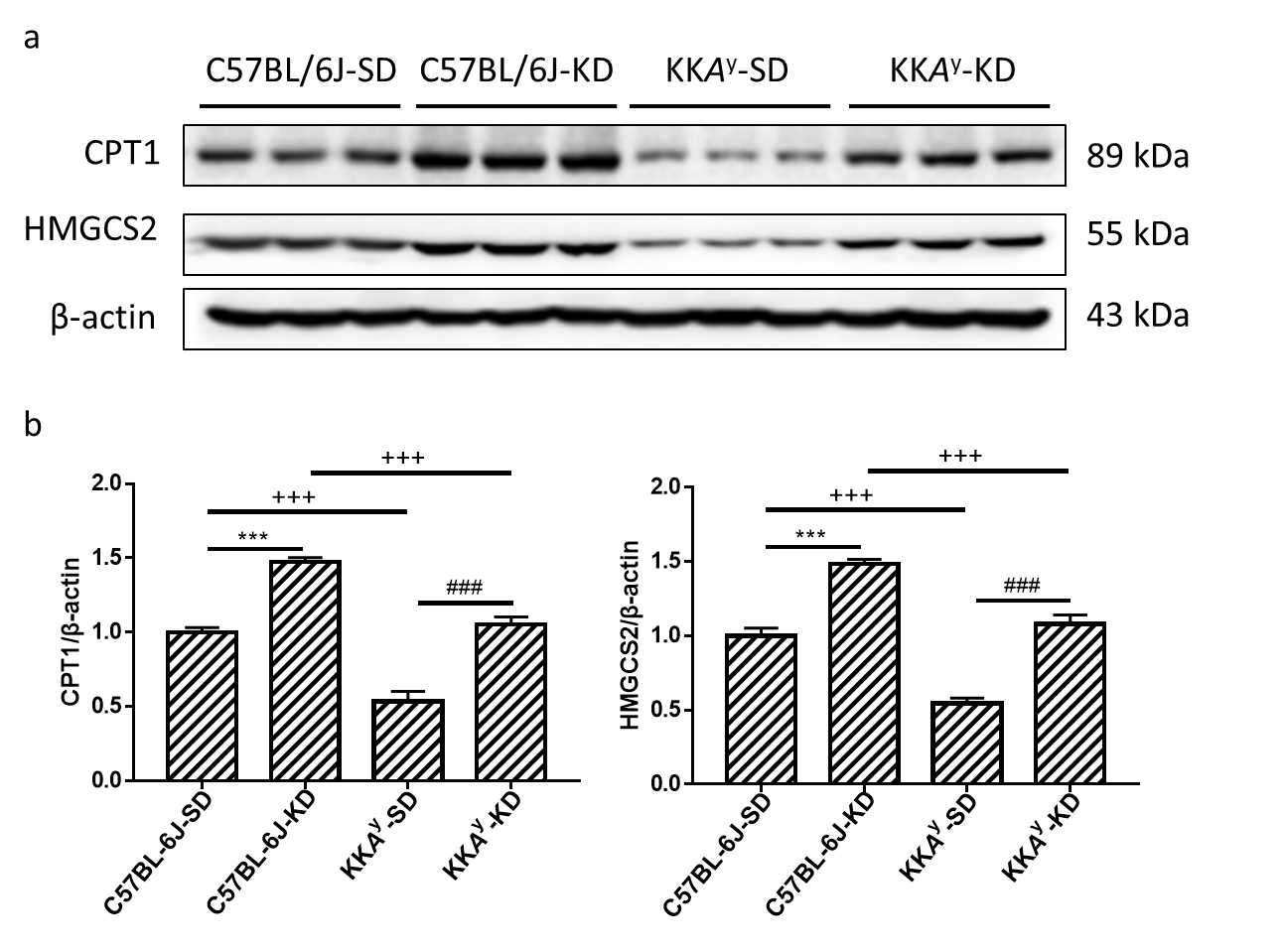
*

**Fig. S4**
